# “Callus Bending Technique” Following Gradual Bone Lengthening for Functional Reconstruction in Cases of Phalangeal Loss Due to Digital Amputation: Two Case Reports

**DOI:** 10.1016/j.jhsg.2024.07.005

**Published:** 2024-08-24

**Authors:** Hiroyuki Gotani

**Affiliations:** ∗ Hand and Microsurgery Center, Osaka Ekisaikai Hospital of Japan Seafarers and Relief Association, Osaka, Japan; † Department of Advanced Medical Engineering for Hand and Microsurgery, Shizuoka Institute of Science and Technology, Fukuroi, Japan

**Keywords:** Bone lengthening, External fixator, Finger trauma

## Abstract

**Purpose:**

The purpose of this study was to introduce the “Callus bending technique” following gradual bone lengthening for functional reconstruction. We report cases of segmental phalangeal loss with replanted incomplete amputation and distal finger amputation.

**Methods:**

Herein, we report two cases to introduce the “callus bending technique”: one in which the “callus bending technique” was adopted after gradual bone lengthening for amputated digital stumps, and the other, a case of incomplete digital amputation, in which defects in the replanted middle phalanges were filled by distraction lengthening of the proximal phalanx, followed by use of the callus bending technique.

**Results:**

The patients could almost touch their palms with the tips of their injured fingers when they flexed their metacarpophalangeal joint of the digit fully. When he extended the metacarpophalangeal joint of the injured fingers fully, the hand looked almost natural, and he could push a table. For the patient with incomplete digital amputation, the distal interphalangeal joint was also reconstructed using the joint surfaces of the distal and proximal phalanges.

**Conclusions:**

Both patients were satisfied with both the functional and cosmetic improvement of their fingers and returned to their work as carpenters. In both cases, an external fixator, the Ilizarov minifixator, was used, as it affords relatively great flexibility.

**Clinical relevance:**

Adoption of this technique involves the use of multiple surgical procedures, but it eventually yields reasonable cosmetic and functional results. We propose the use of this technique as the technique of choice for amputated fingers or severe bone loss due to trauma.

Matev^1^^,^^2^ is well-known for reconstruction of the thumb and has devised various methods for digital distraction lengthening. Sawaizumi reported bone lengthening of an amputated distal phalanx using the Ilizarov minifixator.^3^

In cases of digital amputation associated with traumatic injuries, we have previously reported the healing index of the phalanges and metacarpals.^4^ In addition, we have reported that not only bone lengthening but also three-dimensional expansion of the first web space is possible with the use of the Ilizarov minifixator in patients with post-traumatic contractures of the carpometacarpal joint of the thumb or with post-traumatic soft-tissue injuries.^5^

In this study, based on these experiences, we demonstrated that it is possible to bend calluses manually, much as if they were clay, using a hinge between the units of the Ilizarov minifixator in patients who have undergone gradual lengthening of the proximal phalanx, before bone fusion is fully completed.

The bending angle was determined by reference to the preoperative range of motion (ROM) of the metacarpophalangeal (MP) joint, especially so as to allow sufficient dorsiflexion. In one of the two cases reported herein, with complete amputation of the left middle finger, we bent the callus at its base by gradual lengthening of the proximal phalanges.

In the other case, which was a case of an incomplete amputation, we maintained the finger length using a wire, on the assumption that bone transport occurs in two stages at the time of initial replantation.

After we first confirmed the survival of the amputated finger, we initiated bone transport using the Ilizarov minifixator to restore the finger length. In this case, the flexor digitorum profundus was still present, and before surgery, we planned reconstruction of the distal interphalangeal (DIP) joint with the distal phalanx joint surface and the proximal phalanx joint surface after bone transport.

After completion of bone lengthening of the affected finger and before completion of bone union, we performed callus bending by attaching a hinge between the central and peripheral external fixators across the callus.

Callus bending was performed at the level of the proximal web space rather than in the normal proximal interphalangeal joint. In comparison with joint fixation performed at the proximal interphalangeal level, this method is considered to provide a larger arc and less prominent appearance and facilitate the flexion of the finger to touch the palm.

The purpose of this paper was to introduce cases of replanted fingers with bone defects, etc. where even the MP joint alone can expand motion of the injured finger by gradual bone lengthening and callus bending using the hinge of an external fixator at the base of the proximal phalanx.

(Basic surgical technique for use of the Ilizarov minifixator for gradual bone lengthening)

First, we describe the basic surgical procedure for gradual bone lengthening for amputated stumps of the digits.^4^

We applied the Ilizarov minifixator device (ARATA) to perform lengthening distraction for the affected digit. The Ilizarov minifixator was developed by the Ilizarov Scientific Center in Russia and is composed of a flat-sided rod, nuts, a fixation bolt that moves along the rod, and slotted washers for fixing the wires (Fig. 1).

**Figure 1:**
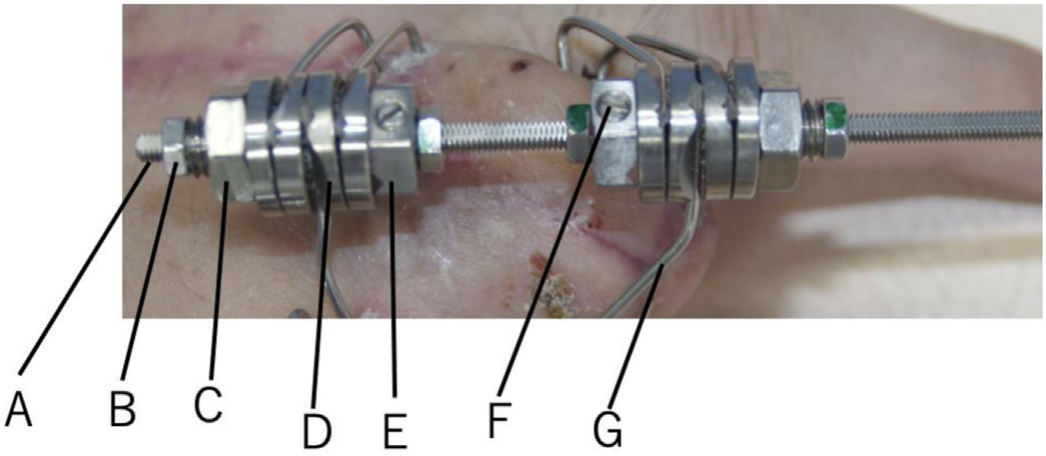
Ilizarov minifixator (top view). **A** Flat-sided rod, **B** nut (M3), **C** nut (M6), **D** slotted washer, **E** fixation-bolt, **F** set screw, and **G** wire.

First, three 1.5-mm wires (Ilizarov wires) were inserted in a crossed pattern in the coronal plane from the radial and ulnar sides of each of the proximal and distal osteotomy sites. The wires were inserted in a crosswise manner to prevent rotation of the fixator and then bent to the midline of the dorsal side of the digits and fixed into the slotted washers on the proximal and distal bolts (Fig. 2). An osteotomy using an osteotome was performed through a small incision on the dorsal side of the digit under fluoroscopic control.

**Figure 2:**
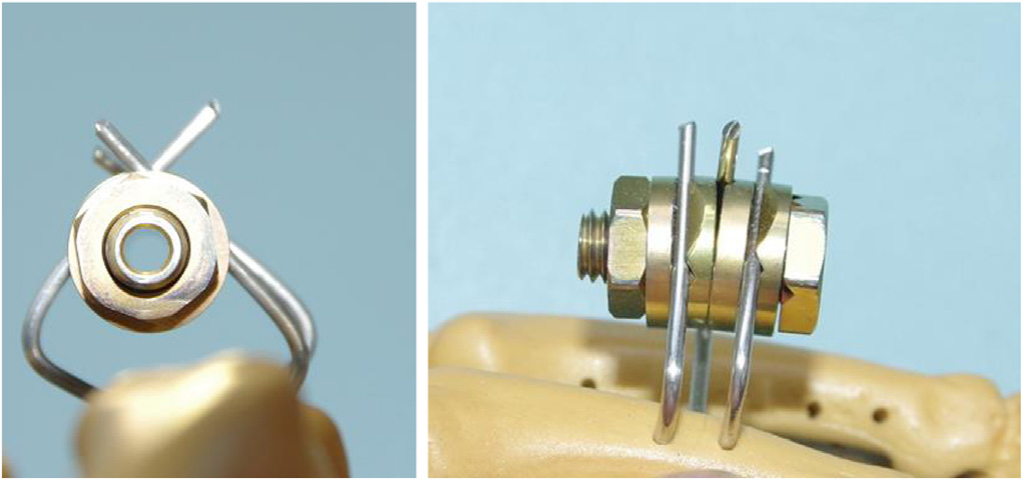
(Left) Coronal view of Ilizarov minifixator without flat -sided-rod. (Right) Lateral view. Crossed wires inserted into the bone to secure fixation and prevent rotation. Three wires were bent to the dorsal side and were fixed into the slotted washer per one unit.

After a 7-day latent period, gradual lengthening was begun at a rate of 0.5 mm per day or one round of nut turning until the desired length was achieved.^2^^,^^3^^,^^6^ The distraction device was removed once the bone consolidation in the gap showed evidence of at least three cortical continuities in anteroposterior and lateral radiographs.^7^^,^^8^

## Materials and Methods

Informed consent was acquired from the patient for the publication of this case report and accompanying images. This study was approved by the institutional review board (20240130) at our institution.

### Case presentation

#### Case 1

**Figure 3:**
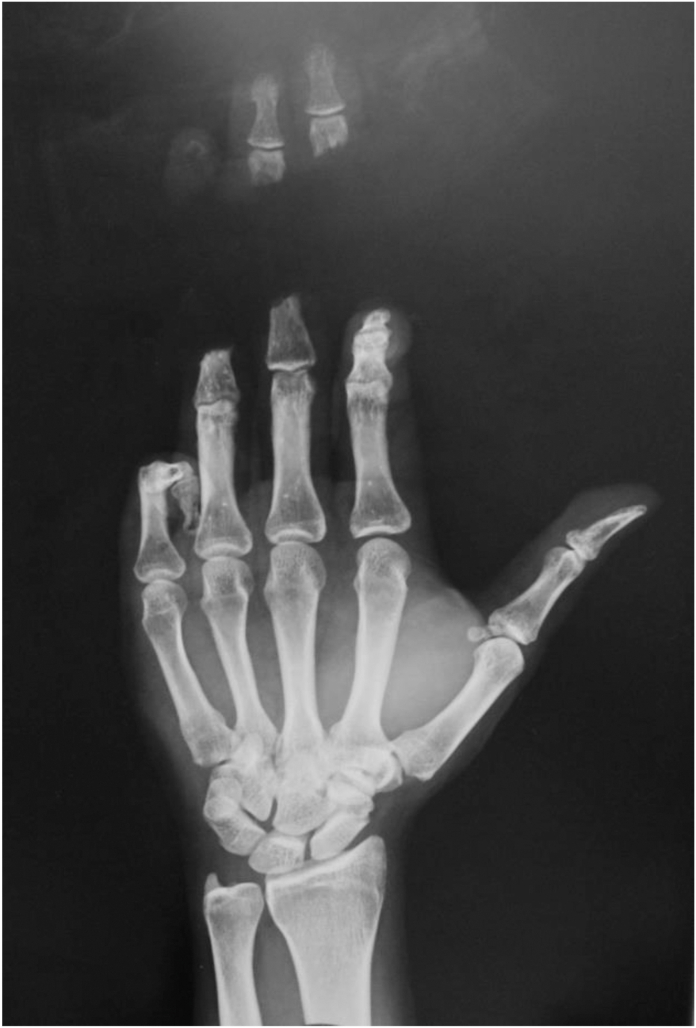
X-ray on admission.

A 47-year-old male patient had suffered an injury with complete amputation, at the middle of the middle phalanges, of the third and fourth fingers of the left hand (Fig. 3); furthermore, the fingertip of his left fifth finger was also completely amputated, with an open fracture of the middle phalanx.

The fourth and fifth fingers were replanted. However, due to development of a contusion of the third finger, we decided to perform bone lengthening after stump plasty.

**Figure 4:**
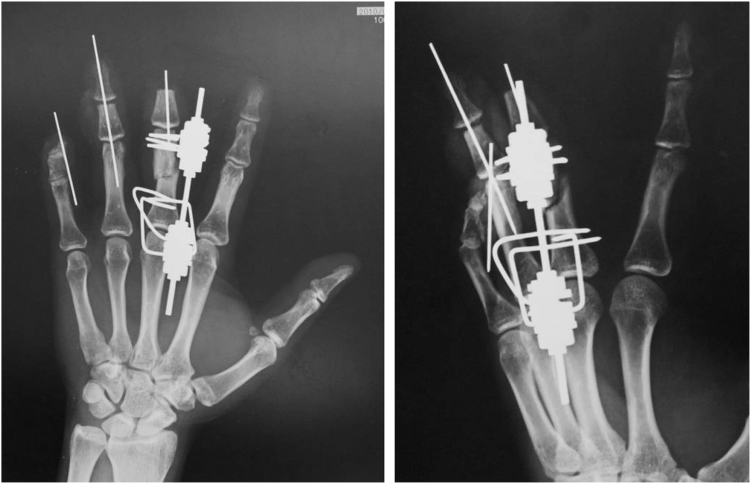
X-ray after application of Ilizarov minifixator.

Three weeks after the injury, when the blood flow was stabilized in the fourth and fifth fingers after the replantation, an Ilizarov minifixator was applied to the middle finger, and an osteotomy of the proximal phalanx was performed (Fig.4). The details of the bone lengthening surgery are described above.

**Figure 5:**
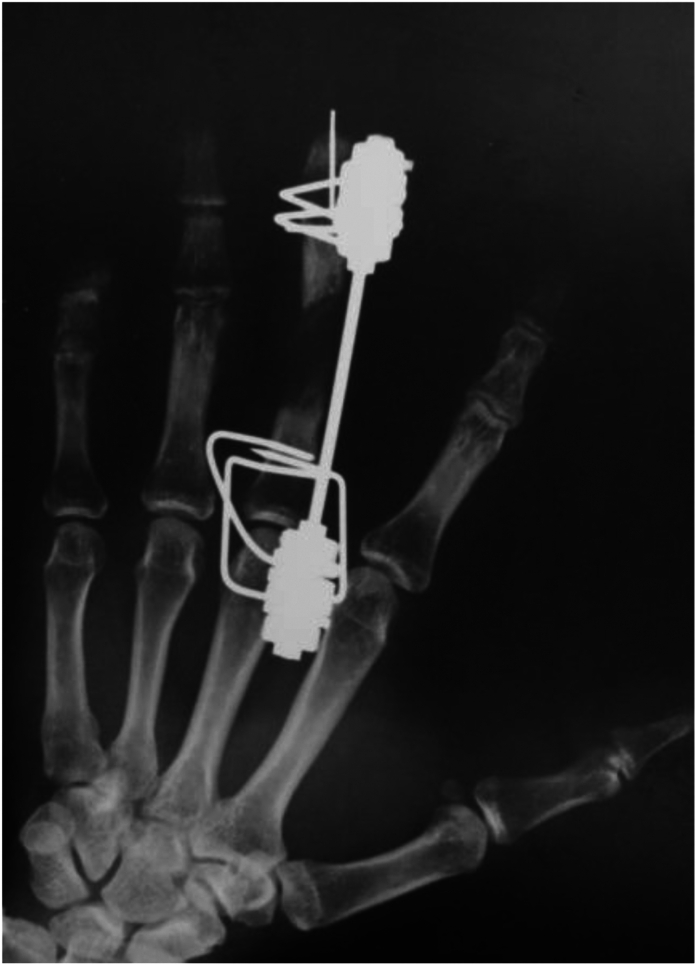
At 50 days from the second operation, bone lengthening to 21mm was achieved.

After application of the Ilizarov minifixator, the gradually distracted bone segment became 21 mm in length over a period of 50 days (Fig. 5).

At 50 days from the second operation, bone lengthening to 21 mm was achieved.

Hinge is shown above the bending site.

**Figure 6:**
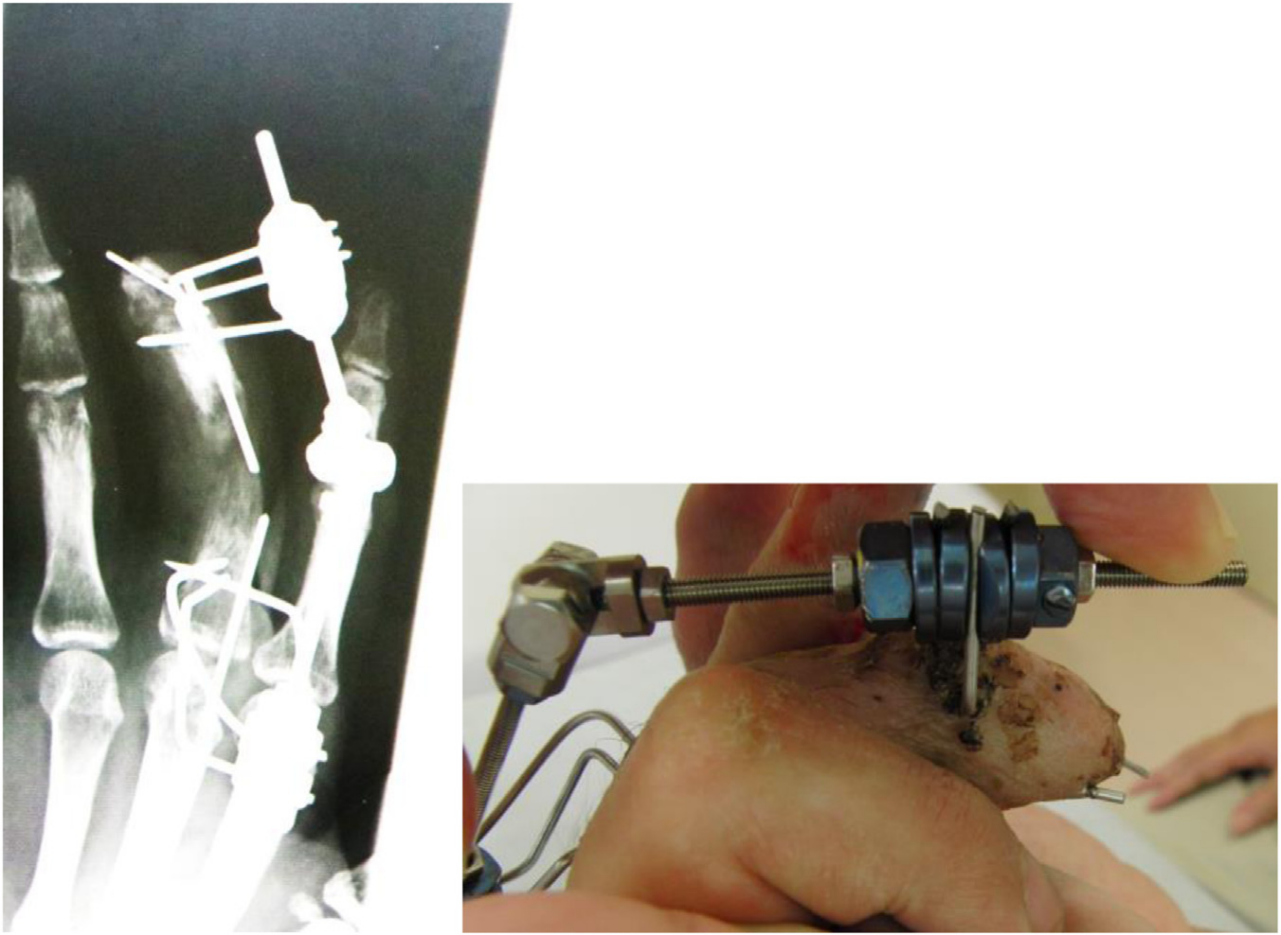
(Left) X-ray shows two wires below the hinge. (Right) Hinge is shown between two M3 units.

Callus formation was observed throughout the gradually distracted section, and we employed the callus bending technique after 85 days from the second operation (Fig. 6, left).

The callus bending site was selected to be a slightly distal portion of the web space, taking into account the following two points: the dorsal aspect of the finger developed a protrusion after the surgery, which needed to be covered by the skin of the web space, and in order to allow the fingertip to come closer to the palm during flexion of the MP joint, we considered that it would be advantageous to select a site closer to the MP joint. Thus, we planned insertion of wires (1.2 mm in diameter) from the proximal and distal sides of the proximal phalanx, so that they could be almost crossed at the distal aspect of the web space, which was planned as the bending site (Fig. 6, right).

Bone fusion was confirmed 65 days after the callus bending, and we performed wire removal.

**Figure 7:**
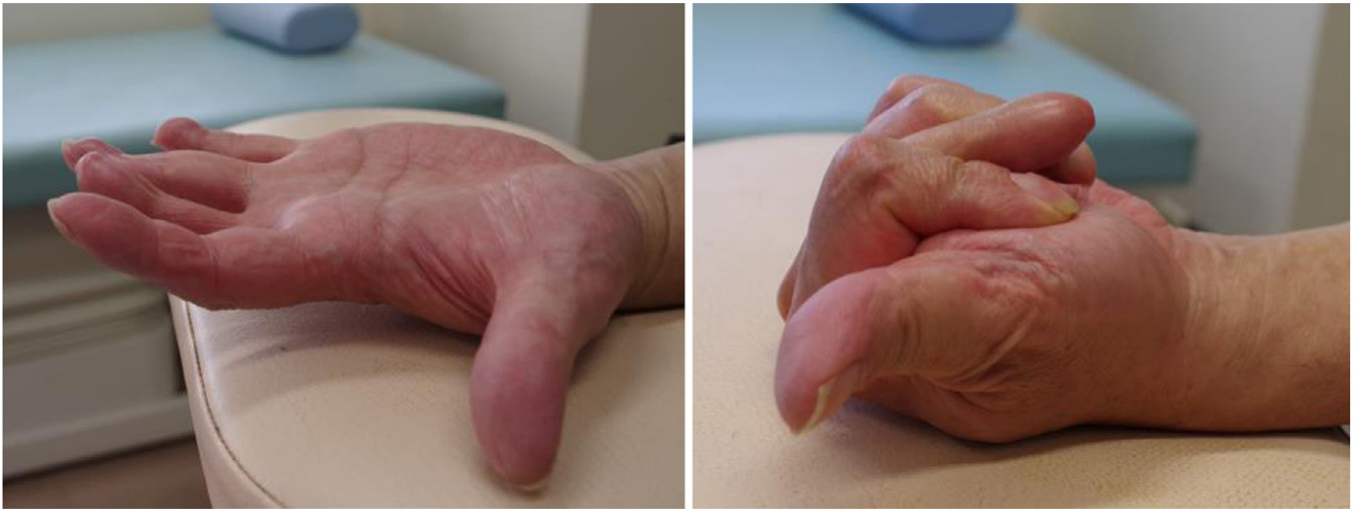
Condition of the hand 5 years after the wire removal. (Left) MP joint extended to almost 30 degrees. (Right) MP joint flexed to almost 80 degrees. MP joint, metacarpo phalangeal joint.

Figure 7 shows the condition of the hand 5 years after the wire removal.

The image on the left side of Figure 7 shows the middle finger in a state of maximum extension (MP joint extended to almost 30 degrees). The image on the right side shows the middle finger in a state of maximum flexion (MP joint flexed to almost 80 degrees). The patient showed a flexion effect of the proximal phalanx at 80 degrees flexion of the MP joint, and the fingertip was positioned 3 cm from the edge of the palm.

**Figure 8:**
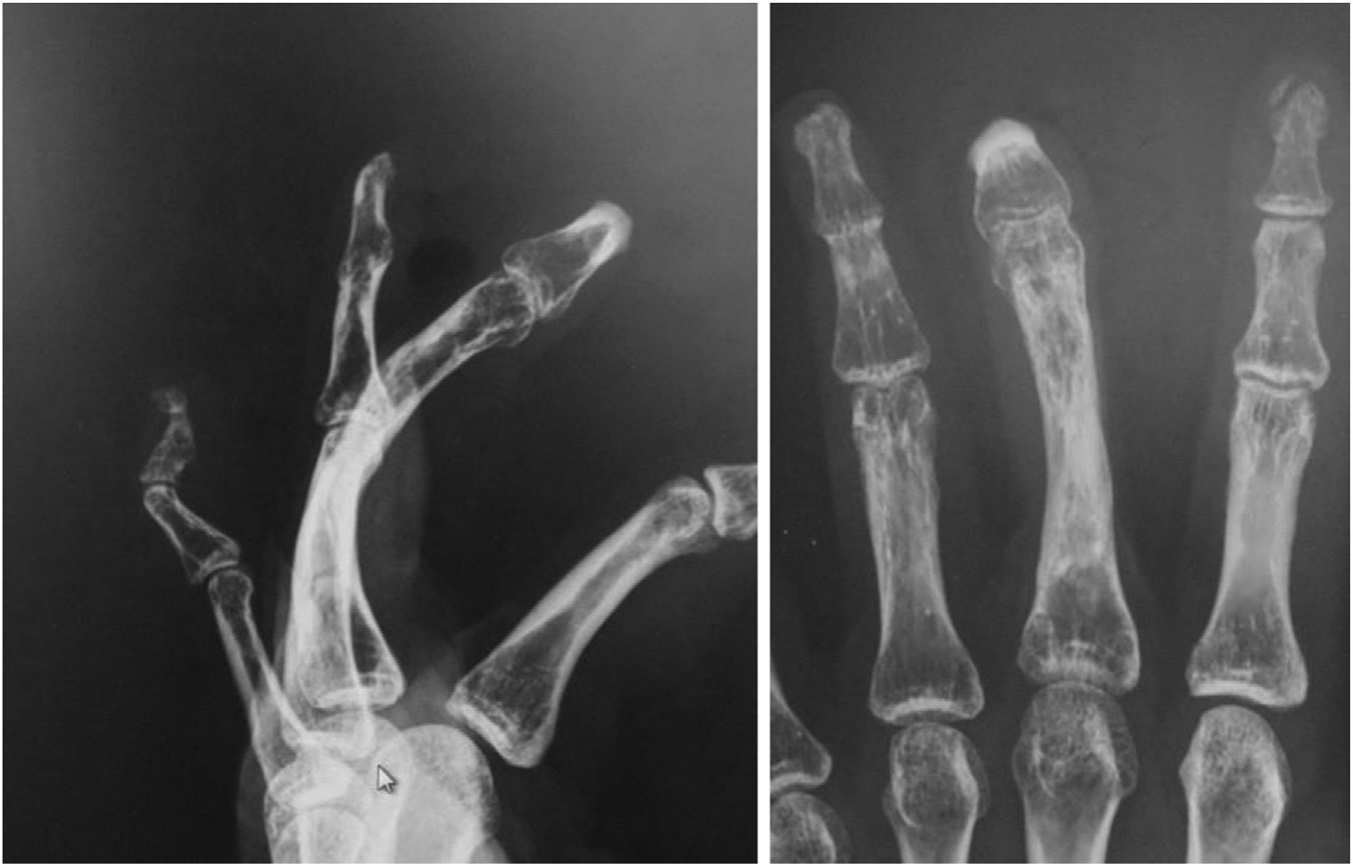
(Left) Lateral view of the X-ray of Case 1, 5 years after the wire removal. (Right) Anterior posterior view of the X-ray.

**Figure 9:**
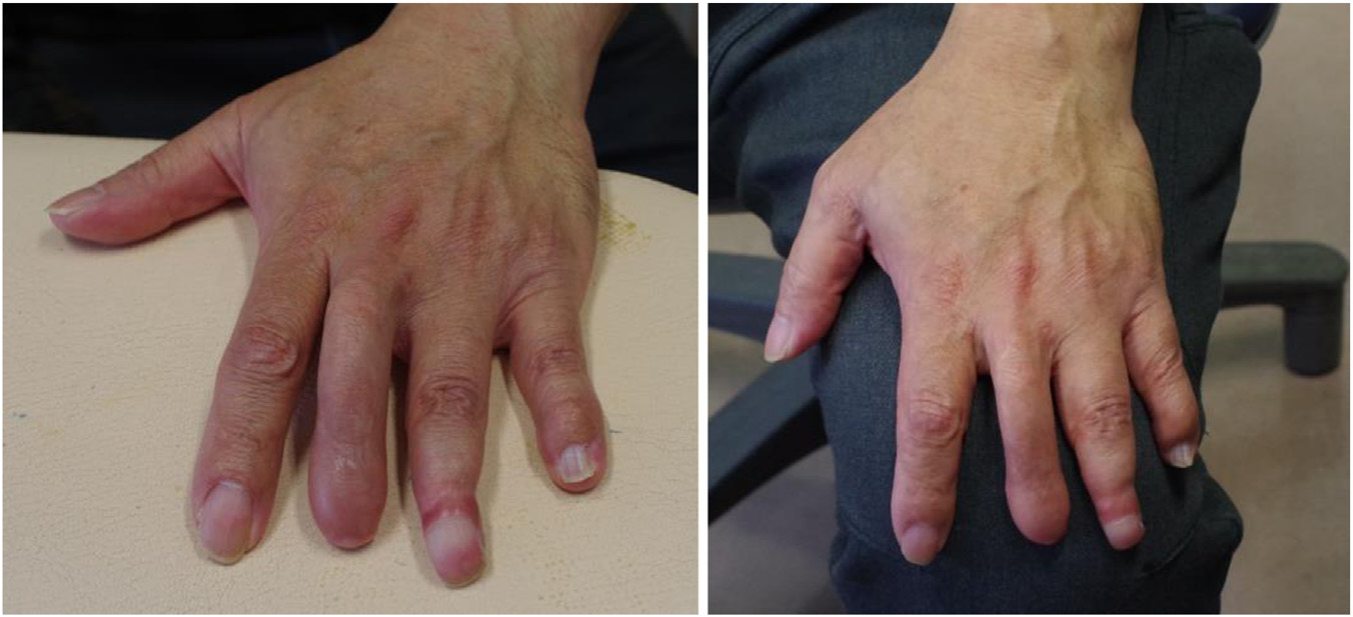
The patient could push the table (left), and his hand looked natural when he placed it on his knee (right).

Figure 8 shows the X-ray at the same time. It shows the lengthened and bent proximal phalanx. The patient could push the table (Fig. 9, left), and his hand looked natural when he placed it on his knee (Fig. 9, right).

#### Case 2

The patient suffered incomplete amputation of the right middle finger while using a drill for drilling holes when the fingers became caught in the rotating drilling machine along with the glove. The tendon of the flexor digitorum profundus remained connected, but the skin was connected only partially on the radial side. The cartilage surfaces of the proximal phalanx head were exposed, but there was no evident damage to the articular cartilage.

**Figure 10:**
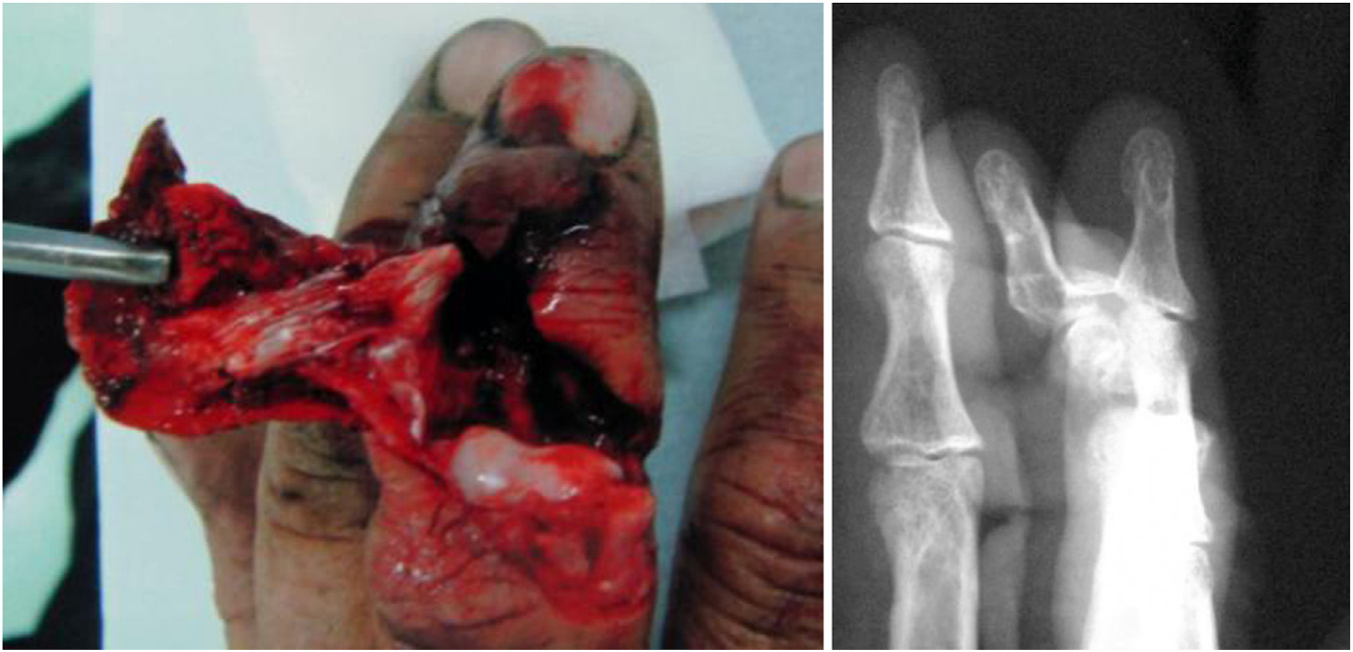
Case 2. (Left) Photograph of the incomplete amputation of the middle finger. (Right) X-ray of the same case. Middle phalange is almost lost.

The extensor tendon was partially attached to the dorsal skin, and no bleeding was observed on needle puncture (Fig. 10).

**Figure 11:**
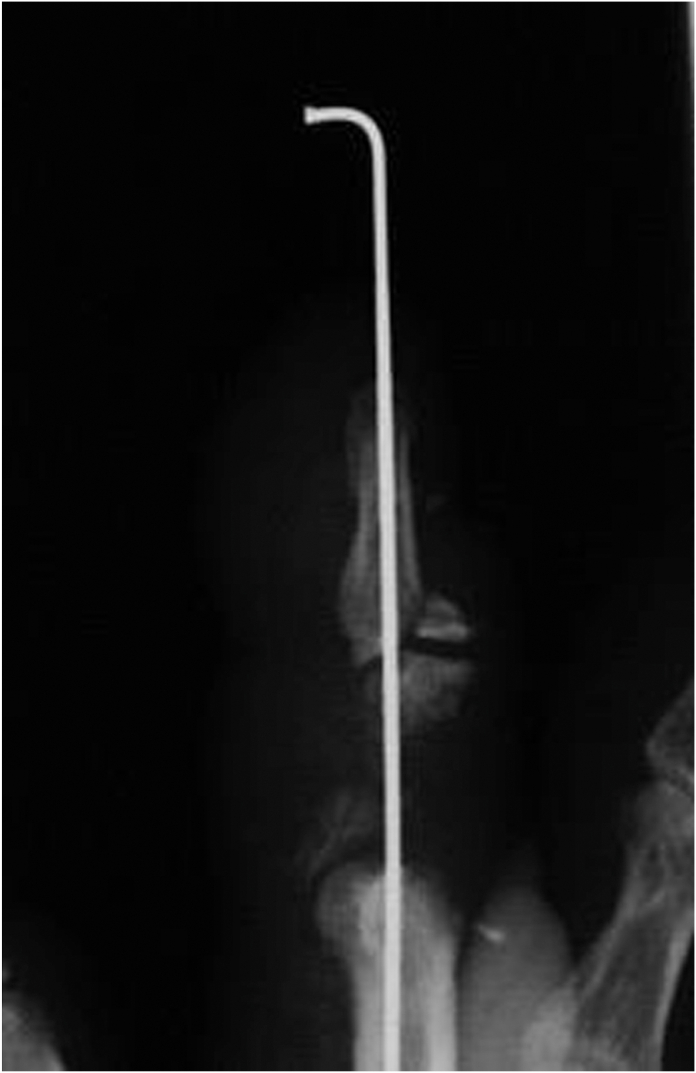
Case 2. The middle finger was replanted and fixated with wire.

Replantation was performed under brachial plexus block anesthesia. The digital arteries were ruptured on the radial and ulnar sides. The digit was shortened by 2 cm to allow anastomosis of both the digital artery and dorsal vein in the unaffected areas. The proximal and distal phalanges were shortened by 2 cm totally and fixed with the wire measuring 1.2 mm in diameter (Fig. 11).

The bilateral digital arteries could be anastomosed at the ends by shortening them by about 2 cm. Although the digital nerves were stretched, the nerves on the radial side were left untreated because of their continuity. On the other hand, the nerves on the ulnar side had been cut and therefore were shortened and sutured.

In this patient, the middle phalanx was missing. Therefore, the following procedure was carried out based on the basic procedure in order to accomplish bone transfer.

The M3 basic unit of the Ilizarov minifixator was fixed to the base of the proximal phalanx with three designated wires measuring 1.2 mm in diameter. One M3 unit was fixed to the replanted distal phalanx, using one 1.2-mm wire in the direction of the long axis and two 1.2-mm wires perpendicular to the axis. In addition, the M3 unit was fixed to the distal end of the proximal phalanx using three wires.

After the entire middle finger was fixed with a 3-mm rod and three units, osteotomy was performed at the base of the proximal phalanx. Bone lengthening（transfer）was performed from the seventh day after the osteotomy.

The nuts used to fix the M3 unit on the rod at both proximal and distal sites were moved by 0.5 mm per rotation per day, which allowed gradual bone lengthening.

**Figure 12:**
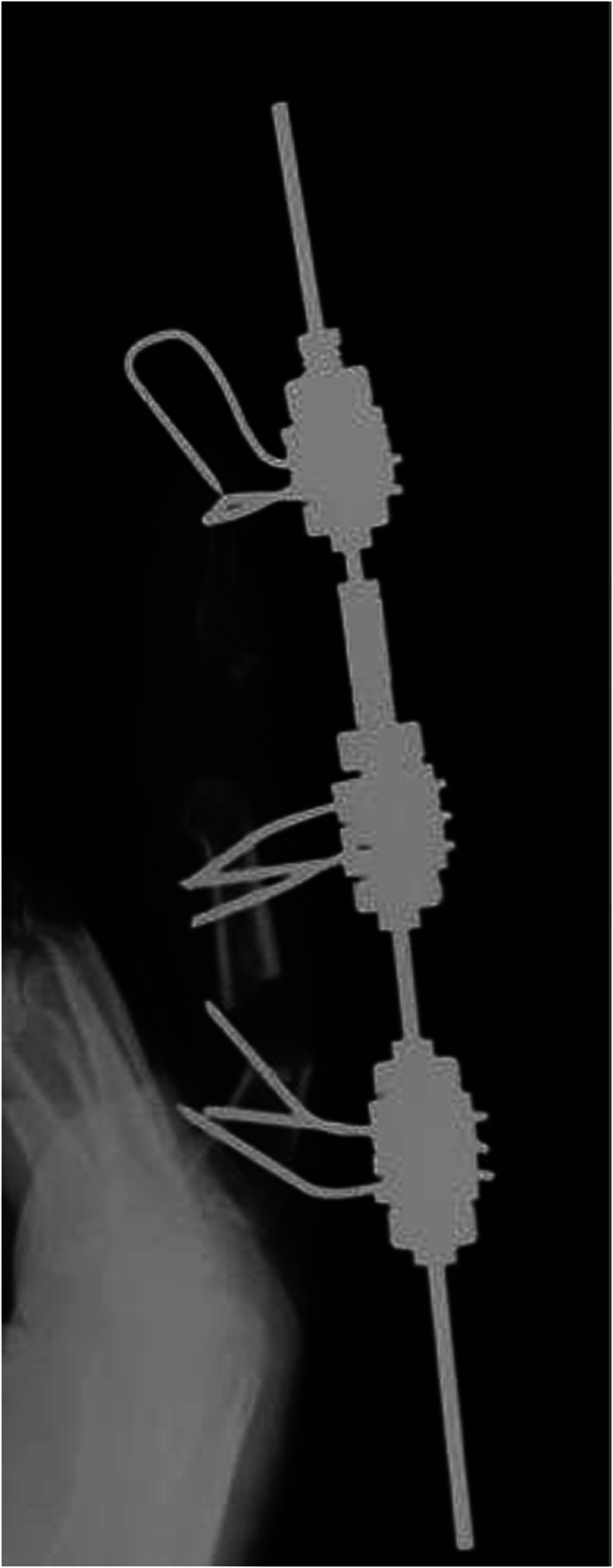
The distal part of the proximal phalanx moved distally by 0.5 mm per day from day 7 after application of the Ilizarov-mini fixator.

Figure 12 shows an X-ray image during bone transfer of the osteotomized proximal phalanx to the distal phalanx.

**Figure 13:**
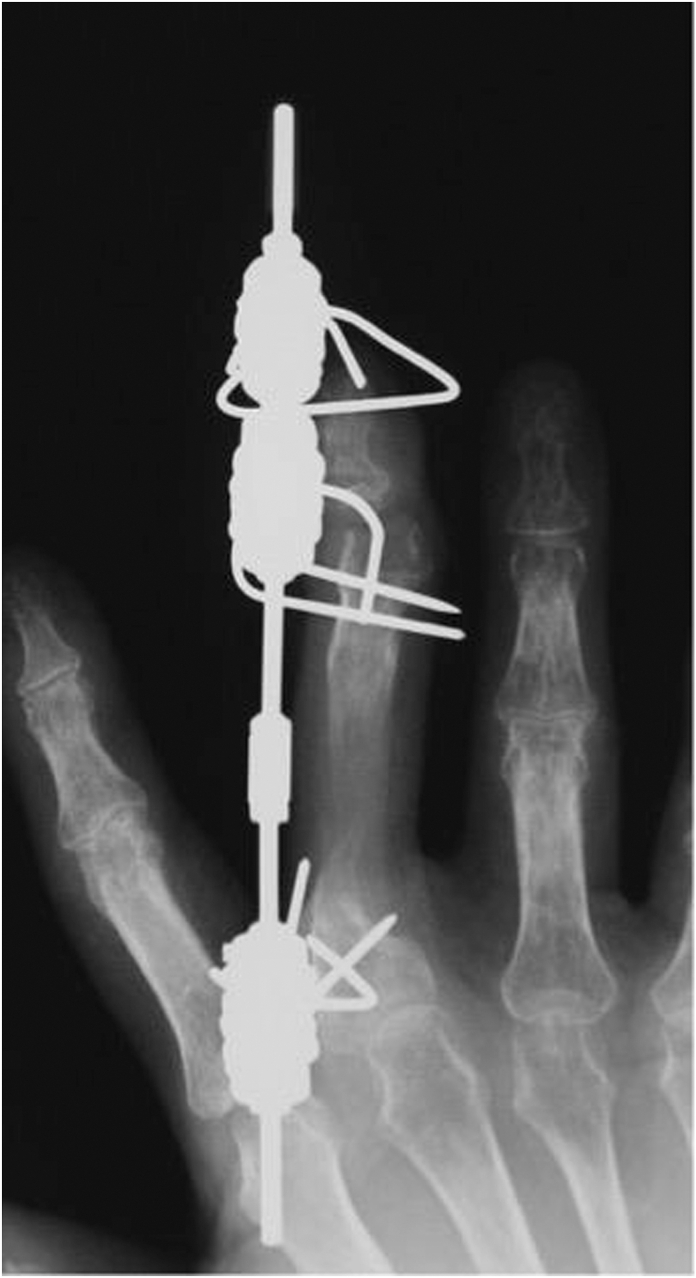
X-ray shows the callus formation after gradual lengthening of the proximal phalanx before callus flexion.

The bone transfer was continued. After the head of the proximal phalanx reached the joint surface of the distal phalanx, callus bending was performed on day 126 after the surgery, after the callus had fully formed, but not yet completely bone united (Fig. 13).

**Figure 14:**
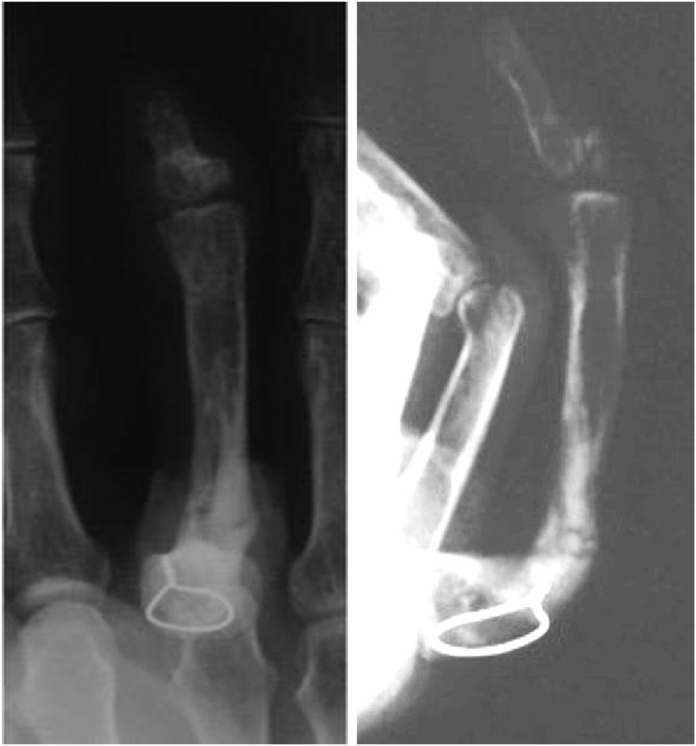
X-ray after bone union, the right image shows the lengthened proximal phalanx bent at an angle of 80°.

**Figure 15:**
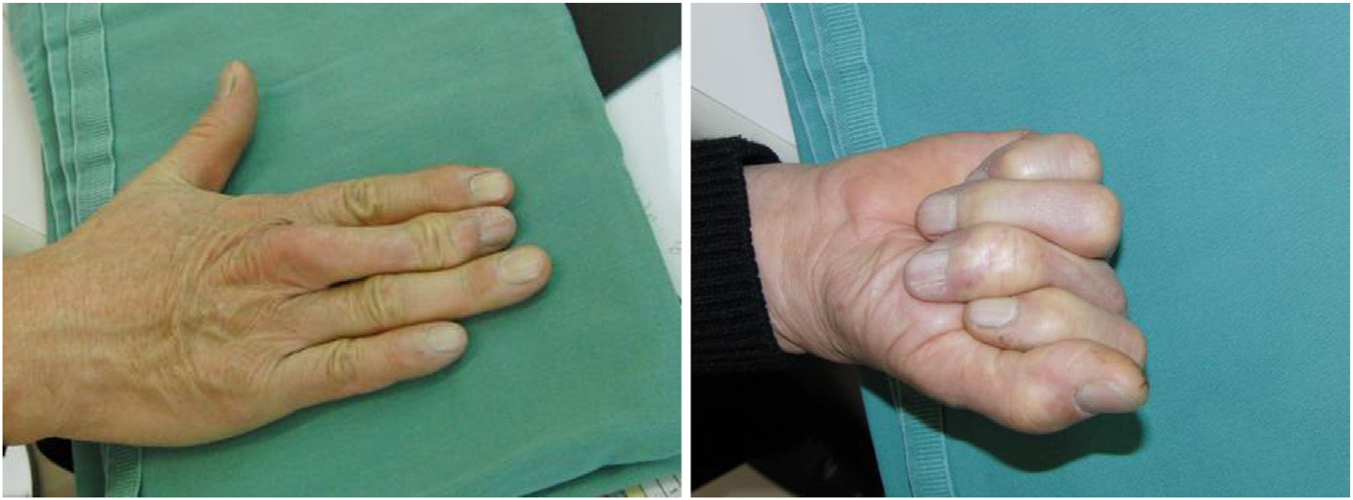
The pushing motion is shown (left). The ROM of the DIP joint formed by the head of the proximal phalanx and joint surface of the distal phalanx (right). DIP joint, distal interphalangeal joint; ROM, range of motion.

Bone fusion was achieved 2 months after the bending, and the wires used for the external fixation were removed (Fig. 14). The pushing motion is shown (Fig. 15, left). Also, the ROM of the DIP joint formed by the head of the proximal phalanx and joint surface of the distal phalanx improved from −20 degrees active extension to 60 degrees active flexion, and it was possible for the patient to bend the finger to touch the palmar surface (Fig. 15, right).

## Discussion

Bone and soft tissue reconstruction with the use of an external fixator is one of the procedures that surgeons need to be skilled in when dealing with severe hand injuries, and previous studies have not reported any neurovascular problems after bone lengthening.

“Formative hand surgery,” proposed by Kuroshima, is a thesis on this kind of procedure devised and developed to deal with individual cases of such injury.^9^

The surgery may be viewed as a surgical procedure aimed at reconstructing crushed hands through a combination of bone and soft tissue extensions achieved with the use of an external fixator. With reference to the skin-extending technique and the technique of reconstruction through leg extension and the use of a tissue expander reported by Bashir,^10^ Kuroshima devised a technique for deforming tissue like clay and reported it as the “formative approach” or “formative surgery.”

The goal of the reconstruction here is not to completely restore the original appearance but to make the injured hand look as close as possible to a normal hand while achieving the necessary functions of the hand using the remaining tissue through tissue extension techniques.

The author often uses the Ilizarov external fixator developed for digits (Arata Co, Ltd), which provides strong fixation while allowing great flexibility. Use of this fixator involves some initial difficulties, such as with the wire bending technique. However, once the technique for installing the basic M3 and M4 units is mastered, it becomes possible to connect various devices using a wide variety of parts. Therefore, this device is very useful not only for post-traumatic contractures but also for bone lengthening.

In patients with joint contractures, the Ilizarov external fixator allows the soft tissues to stretch gradually while providing a traction force to the joint. Thus, it can simultaneously achieve soft tissue expansion and joint dissection.

In the cases reported herein, we were able to manually bend the callus located proximal to the proximal phalanx by applying the basic bone lengthening technique using the Ilizarov minifixator. This technique requires the use of a hinge placed between the external fixation units and crossing the wires at the site of the bending osteotomy. The osteotomy angle was 45 degrees in case 1 and 80 degrees in case 2. This was possible because an adequate ROM was achieved at the MP joint during the proximal phalanx lengthening. In particular, we think it is important that the patient can extend MP joint almost same as healthy side before “callus bending” surgery to acquire enough ROM after surgery. More specifically, if 80 degrees flexion and about 30 degrees of extension can be maintained at the MP joint before callus bending, the fingertip can be brought much closer to the palm after callus bending. In addition, the natural pushing motion was also restored in the patients.

In the time course of this procedure, most patients came to outpatient clinic to have an examination of the fixator at least one time per week. As it takes a long time to achieve this procedure for waiting bone consolidation, there is a possibility that we will try to do the bone graft to enhance bone consolidation after this technique or try to remove the fixator to change to the internal fixation if the patient hopes to shorten the time of application of external fixator.

## Conclusions

Adoption of this technique involves the use of multiple operative procedures, but it eventually yields reasonable cosmetic and functional results. We propose the use of this technique as the technique of choice for amputated fingers or severe bone loss due to trauma.

## Conflicts of Interest

No benefits in any form have been received or will be received related directly to this article.

## References

[1] Matev I.B. (1970). Thumb reconstruction after amputation at the metacarpophalangeal joint by bone-lengthening: a preliminary report of three cases. J Bone Joint Surg Am.

[2] Matev I.B. (1989). The bone-lengthening method in hand reconstruction: twenty years’ experience. J Hand Surg Am.

[3] Sawaizumi T., Ito H. (2003). Lengthening of the amputation stumps of the distal phalanges using the modified Ilizarov method. J Hand Surg Am.

[4] Kanchanathepsak T., Gotani H., Hamada Y. (2020). The effectiveness of distraction lengthening in traumatic hand amputation with ilizarov mini fixator. Injury.

[5] Salimi H., Gotani H., Tanaka Y. (2022). First web space plasty using Ilizarov mini fixator in patients with complex hand injuries. Orthoplastic Surg.

[6] Toh S., Narita S., Arai K., Nakashima K., Tsubo K. (2002). Distraction lengthening by callotasis in the hand. J Bone Joint Surg Br.

[7] Seitz WH, Shimko P., Patterson R.W. (2010). Long-term results of callus distraction-lengthening in the hand and upper extremity for traumatic and congenital skeletal deficiencies. J Bone Joint Surg Am.

[8] Bozan M.E., Altinel L., Kuru I., Maralcan G., Acar M., Durmaz H. (2006). Factors that affect the healing index of metacarpal lengthening: a retrospective study. J Orthop Surg (Hong Kong).

[9] Kuroshima N., Ohe T., Inoue H. (1996). Proposal for new formative hand surgery with using external fixators. J Jpn Soc Surg Hand.

[10] Bashir A.H. (1987). Wound closure by skin traction: an application of tissue expansion. Br J Plast Surg.

